# Supplementary material for: Leveraging social media in digital scholarship: Perspective from developing country students
Source: PLoS One. 2023 Sep 22;18(9):e0291334. doi: 10.1371/journal.pone.0291334 (PMC10516410; doi:10.1371/journal.pone.0291334)
Supplement: Attachment — Submitted filename: Point-by-point response to the reviewers feedback.doc [file pone.0291334.s001.doc]

Point-by-point response to the reviewers’ feedback

| Page | Comments | How they have been addressed |
| --- | --- | --- |
|  | **Editor** |  |
| Page 8 | The "Empirical Review" section requires some revision, as it is currently written as a serial summary instead of a synthesis of the literature that helps us to understand the relevant themes and highlights what we already know about this topic, what is unknown, and why it is important to address that knowledge gap. | The empirical review section has been revised to help understand the relevant themes in the study and why it is important to address that knowledge gap in pages 8 to 10. |
|  | Please amend your authorship list in your manuscript file to include all the authors. | The authorship list has been amended to include all authors. |
|  | Please amend your list of authors on the manuscript to ensure that each author is linked to an affiliation. | List of authors has been amended to include each author affiliation. |
|  | Please provide additional details regarding participant consent. In the ethics statement in the Methods and online submission information, please ensure that you have specified. | Participants written content statement has been added to the methodology part. |
|  | **Reviewer 1** |  |
|  | It would be fascinating to see another survey (and possibly a follow-up article) discussing the findings after the improvements mentioned, based on student feedback, are implemented for some time | The authors will consider this suggestion and work on another article considering this suggestion. |
| Page 15 | It would be helpful to have some clarification on how the data found in Table 3 was gathered. Was this from the data you gathered in the survey, or was it gathered by some other means? | Using the quantitative data gathered from the postgraduate students, the results for the relationship between students’ satisfaction and behavioural intentions to use e-library support services were presented in Table 3. |
|  | **Reviewer 2** |  |
| Page 1 | Consider changing the title to: Students' satisfaction with e-library services: perspectives of postgraduate distance education students | This has been adjusted appropriately on page 1 and 5. |
| Page 3 | The Introduction does not offer a good justification for the study. What exactly is the problem being addressed in the study? | Part off the introduction has been rewritten to offer good justification for the study in pages 3 and 4. |
| Page 4 | The problem statement is very winding and doesn't clearly bring out why the perspective of postgraduate distance education students should be sought regarding an investigation into the satisfaction of students with e-library services | The problem of study part has been rewritten to show how postgraduate distance education students should be sought regarding an investigation into the satisfaction of students with e-library services in pages 4 and 5. |
| Page 6 | The authors could not give any reason how the Innovation diffusion theory is important for a study of this nature | The authors have now given reasons why innovation diffusion theory is important for this study in page 6. |
| Page 11 | The authors only mentioned using the mixed methods approach for the study without justifying why they chose this approach as against a quantitative or qualitative research approach | The authors have justified why they used the mixed methods approach as against a quantitative or qualitative research approach. |
| Page 11 | Merely stating that the mixed methods approach "allowed for the collection of both quantitative and qualitative data" is not a good enough justification | The researchers have given justification for using adopting mixed methods approach in page 11. |
| Page 16 | The presentation does not indicate that the study is a mixed methods research. The use of the mixed methods is not demonstrated in the discussions and in the results - presented like a purely quantitative study; there was no direct quotations to support the findings. | Direct quotations have been provided to support the qualitative part in pages 16 and 18. |
| Page 10 | The authors did not give any reason for the choice of postgraduate distance education students for this study. | The reasons for using postgraduate distance education students have been explained in pages 10 and 11. |
| Page 10 | How does the perspective of postgraduate distance education students help in understanding the issue being investigated? | The reason why postgraduate distance education students were used has been explained in pages 10 and 11. |
| Page 12 | A response rate of 99.5% is unusually high. It would have been helpful if the authors could give some explanation to this high response rate | A period of two months was used for data collection. This help the researchers to convince almost all of the participants to participate in the study. |
| Page 13 | Some extraneous information which have no bearing on the study are introduced into the work! The information on the moderating role of gender and education in examine factors | It is true, however, the researchers wanted to give information on some of the demographic characteristics of the participants. |
| Page 11 | Also, first time usage of certain terms without explanation - for instance under Methodology, the authors assumed that all readers will know what CoDe means | This have been addressed in page 11. |
