# Supplementary material for: Leveraging social media in digital scholarship: Perspective from developing country students
Source: PLoS One. 2023 Sep 22;18(9):e0291334. doi: 10.1371/journal.pone.0291334 (PMC10516410; doi:10.1371/journal.pone.0291334)
Supplement: Attachment — Submitted filename: Re-review SM_DS.docx [file pone.0291334.s002.docx]

**Re-Review**

**Title: Leveraging social media in digital scholarship: Perspective from developing country students**

1. There has been some substantial revision to the original paper, For example:
   a. The abstract has been improved.
   b. The purpose has been clarified.
   c. Key concepts have been explained.
   d. The language has been improved
   e. Appropriate citations have been provided
2. However there are still issues to consider. For example:
   a. Whilst the paper adopts the Diffusion of Innovation (DOI) Theory, it is not clear how it has been used in this study [neither does it reflects in the Research Questions, Analysis or Discussions]. For instance, you may change the theory for a theory that captures your key constructs or modify the constructs entirely to reflect your selected theory.
   b. Whilst the purpose has been clarified, there is the need for the research questions to be related and help contribute to the purpose.
3. Publishability:

The paper can be published upon the address of the highlighted concerns
