# Supplementary material for: Leveraging social media in digital scholarship: Perspective from developing country students
Source: PLoS One. 2023 Sep 22;18(9):e0291334. doi: 10.1371/journal.pone.0291334 (PMC10516410; doi:10.1371/journal.pone.0291334)
Supplement: Attachment — Submitted filename: A rebuttal letter.doc [file pone.0291334.s003.doc]

| Comments | How they have been addressed |
| --- | --- |
| **Reviewer 2** |  |
| Whilst the paper adopts the Diffusion of Innovation (DOI) Theory, it is not clear how it has been used in this study [neither does it reflects in the Research Questions, Analysis or Discussions]. For instance, you may change the theory for a theory that captures your key constructs or modify the constructs entirely to reflect your selected theory. | The authors get your concern why the Diffusion of Innovation (DOI) theory does not reflect in the research questions. Research question one sought to examine the knowledge level of students in the use of social media as a digital scholarship. The DOI theory underscore the use of technology features like compatibility, complexity, trialability and observability which have some linkage with users’ knowledge level. Also, additional information have been provided on the discussion part to link thhe DOI theory to the study. |
| Whilst the purpose has been clarified, there is the need for the research questions to be related and help contribute to the purpose. | This has been done. |
| **Reviewer 3** |  |
| The paper lacks the fundamental for scale development. The questions used to solicit responses from participants lacks the basics statistical foundation for scale development. The 12 item-scale need to go through a "semantic analysis" , "Expert panel analysis" and validation before it can be used. | This has been done. |
| In my previous comment I ask about the I-CVI threshold that was set for the questions (items). | The authors have gave information on the Cronbach coefficient was higher than 0.5, it means that the internal consistency of the items in the questionnaire was satisfactory. |
